# “Hand down, Man down.” Analysis of Defensive Adjustments in Response to the Hot Hand in Basketball Using Novel Defense Metrics

**DOI:** 10.1371/journal.pone.0114184

**Published:** 2014-12-04

**Authors:** Peter Csapo, Markus Raab

**Affiliations:** 1 Institute of Psychology, German Sport University, Cologne, Germany; 2 School of Applied Science, London South Bank University, London, United Kingdom; University of Westminster, United Kingdom

## Abstract

The hot-hand phenomenon, according to which a player’s performance is significantly elevated during certain phases relative to the expected performance based on the player’s base rate, has left many researchers and fans in basketball puzzled: The vast majority of players, coaches and fans believe in its existence but statistical evidence supporting this belief has been scarce. It has frequently been argued that the hot hand in basketball is unobservable because of strategic adjustments and defensive interference of the opposing team. We use a dataset with novel metrics, such as the number of defenders and the defensive intensity for each shot attempt, which enable us to directly measure defensive pressure. First, we examine how the shooting percentage of NBA players changes relative to the attributes of each metric. We find that it is of lesser importance by how many defenders a player is guarded but that defensive intensity, e.g., whether a defender raises his hand when his opponent shoots, has a larger impact on shot difficulty. Second, we explore how the underlying metrics and shooting accuracy change as a function of streak length. Our results indicate that defensive pressure and shot difficulty increase (decrease) during hot (cold) streaks, so that defenders seem to behave according to the hot-hand belief and try to force hot players into more difficult shots. However, we find that shooting percentages of presumably hot players do not increase and that shooting performance is not related to streakiness, so that the defenders’ hot-hand behavior cannot be considered ecologically rational. Therefore, we are unable to find evidence in favor of the hot-hand effect even when accounting for defensive pressure.

## Introduction

The hot-hand phenomenon in sports has sparked many debates about discrepancies between perception and reality. Most basketball coaches, players and fans believe in the hot hand, according to which a player’s performance is expected to be elevated following three or more consecutive hits, but convincing empirical evidence which statistically supports this belief has been sparse until recently (for an overview, see Bar-Eli, Avugos, & Raab [1]). However, nearly all studies examining the hot-hand effect in basketball have made a crucial simplifying assumption by excluding the effect of defensive pressure. While the potential importance of defense with respect to the hot hand has been acknowledged frequently, traditional statistics, i.e., box score and play-by-play data, in basketball have not provided any metrics which could directly measure the impact of defensive pressure.

In this study, we make use of a dataset which includes several novel defensive metrics, such as the number of defenders guarding a shot and the defensive intensity, and thus provide a new perspective on how defensive pressure may affect the hot hand. In basketball, a commonly used expression is “hand down, man down,” meaning that if a defender does not contest a shot by raising his hand, the shooter is likely going to make the shot. The underlying dataset is the only one to account for this important aspect. Consequently, we aim to explore whether defenders act according to the hot-hand belief and whether their actions may lead to the unobservability of the hot hand in team sports. The goal of the current work is to answer two research questions: (1) How does the field goal percentage (FG%) of NBA players change based on the attributes of each variable, e.g., whether a player is guarded by one, two or three defenders? (2) How do these metrics change in relation to streakiness and how do they affect the FG% during hot and cold streaks?

The hot-hand debate has been ongoing since Gilovich, Vallone and Tversky [2] first examined the phenomenon using three different approaches, which involved field goal and free throw shooting data from NBA games and a shooting experiment with varsity college players. None of these analyses yielded evidence in favor of the hot hand, leading them to conclude that the phenomenon did not exist. The widespread belief in the hot hand has been demonstrated frequently in the literature (e.g., Raab, Gula, & Gigerenzer [3]) but until the beginning of this decade, most evidence for its existence has come in sports without direct defensive interference, such as horseshoe pitching (Smith [4]).

However, the increased availability of large-scale datasets in sports and novel statistical approaches have fueled a recent surge in hot-hand research and finally provided more convincing evidence for the hot hand (see Iso-Ahola & Dotson [5] for an overview). Specifically, researchers examined why studies have failed to detect the hot hand in basketball, as one of the most frequently cited arguments is the lack of statistical power of the utilized tests. For instance, Arkes [6] used a multivariate framework with individual fixed effects and found that NBA players were significantly more likely to hit their second free throw after having hit the first one. Similarly, Yaari and Eisenmann [7] found that the conditional probability of hitting the second free throw increased by 1.4 to 4.6% for NBA players if the first free throw resulted in a hit.

Furthermore, a shift in focus towards the examination of behavioral consequences stemming from the hot-hand belief has taken place in the literature over the last decade. For instance, Burns [8] showed that a team which consistently passes the ball to a hot player may score slightly more points. According to the concept of ecological rationality, a belief is evaluated relative to the environmental structure and the hot-hand belief can be ecologically rational in the light of these results. Specifically, it is of primary importance whether behavior based on a certain belief leads a decision maker to achieve his aspiration level and not whether this belief is normative (Gula & Raab [9]). In the context of basketball, hot-hand behavior can be ecologically rational as long as it leads a team to score more points or allow fewer points than the opposing team.

In contrast to Burns [8], who examined hot-hand behavior on offense, Aharoni and Sarig [10] evaluated whether defenders act according to the hot-hand belief. Specifically, they used offensive metrics to approximate the impact of streakiness on defensive pressure and found a hot-hand effect as well as an increase in shot difficulty during hot streaks. The authors hypothesized that the findings were due to defensive pressure as opposed to increased self-confidence.

Studies as early as Gilovich et al. [2] acknowledged the potential importance of defensive pressure by stating that “once a player has made one or two shots, the opposing team may intensify their defensive pressure on that player and ‘take away’ his good shots” (p. 303) but until recently there has been a lack of adequate metrics to capture defensive behavior. However, significant advances have been made in sports analytics over the last years (Alamar & Mehrotra [11]). For instance, Bocskocsky, Ezekowitz and Stein [12] used motion-tracking technology to estimate shot difficulty and defensive intensity by looking at the distance between the shooter and the closest defender as well as the height differential between the two players. Similarly to the above-mentioned studies, they found that shot difficulty increased and that players performed slightly better during hot streaks.

The current study is divided into two phases based on the aforementioned research questions. First, we will present the new defensive metrics and analyze the effect of selected attributes of each metric, e.g., whether a shot occurred from the high post or three-point range, on the FG% of NBA players. Second, we will examine how the metrics and the shooting performance change as a function of streakiness. Overall, we find that defenders increase their pressure on players who have hit several consecutive shots and behave according to the hot-hand belief. However, after controlling for shot difficulty, we do not find the performance of presumably hot players to be elevated, so that the hot-hand behavior of defenders cannot be considered as adaptive.

## Phase 1: How Does the FG% of NBA Players Change in Relation to the Underlying Metrics?

### Method

The study was approved by the university’s ethics committee. The dataset was provided to us by Vantage Sports and included data from 666 NBA games from the 2011–12 to 2013–14 seasons. In total, 94,056 shot attempts were fully coded in the dataset and used for the following analysis in Phase 1. Vantage Sports generates its data through algorithms and human analysis, and accuracy is ensured through an inter-rater-reliability system. An audit attested an accuracy level of 99.7%, making the data more accurate than official play-by-play data. We also compared randomly selected data to the official play-by-play data and found that the information was matching. We were given the raw data by Vantage Sports in the .json file format and wrote a software application which filtered and converted the data into Excel files. The filtering was necessary because the dataset included a vast amount of game data and we only utilized variables associated with defensive pressure, shooting accuracy and hot-hand behavior in our analysis. Furthermore, it is notable that the dataset included missed shots where the shooter was fouled, whereas other datasets only include fouled shots if the shot is made. Therefore, the reported shooting percentages are somewhat lower. In Phase 1, the dataset was filtered based on the attributes of each variable to calculate the respective number of hits and misses.

#### Shot type

Shot attempts were divided into 14 categories and we clustered similar shot types, e.g., fade-away jumper, turnaround fade-away jumper to the left, turnaround fade-away jumper to the right, turnaround jumper to the left, and turnaround jumper to the right into one category to render the analysis and the description of our results more clear-cut. Specifically, we used the following five categories: dunks and layups, fade-away and turnaround jumpers, floaters, jumpers, and hook shots.

#### Dribbles

This metric counts the number of dribbles a player has taken before a shot. We hypothesize that a player who takes many dribbles is likely to have to create his own shot, which tends to be more difficult. This variable was divided into five categories ranging from no dribble to four or more dribbles before a shot attempt.

#### Remaining time on shot clock

If a relatively large number of seconds is left on the shot clock, a shot likely occurred during a fast-break or following an offensive rebound, both of which normally result in easier shots. In contrast, shot attempts close to the expiration of the shot clock tend to result from a possession in which the defense did not allow the opposing team to score easily.

#### Shot location

**Figure 1:**
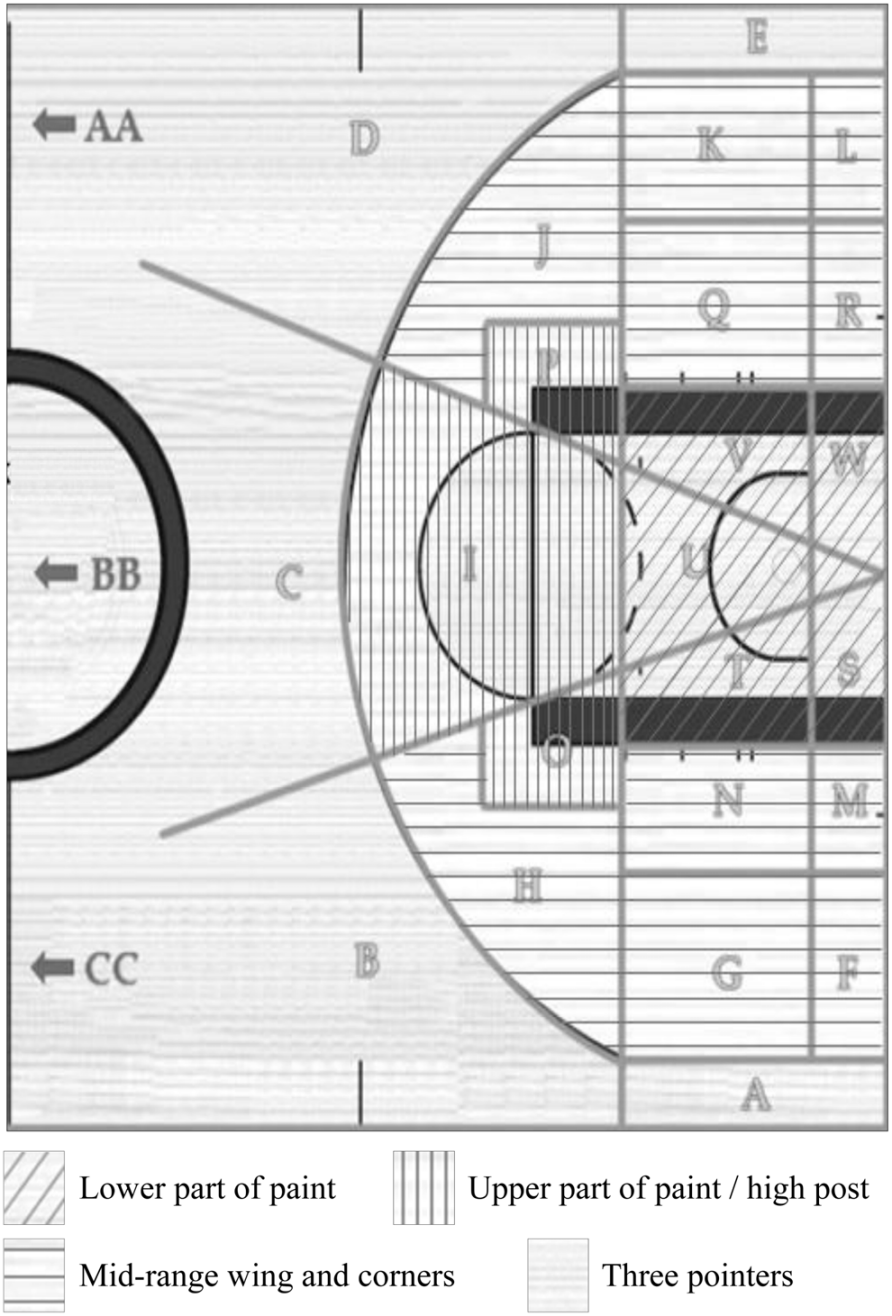
Segmentation of the Different Shot Locations Based on the Vantage Sports Dataset (Indicated by Letters) and the Utilized Clusters in this Analysis (Indicated by the Shading and the Legends).

Shot difficulty tends to increase with distance (see Bocskocsky et al. [12], Neiman & Loewenstein [13], and Attali [14]). The underlying dataset accounted for shot location by symmetrically dividing the basketball court into 26 sections. We grouped the sections into four categories to provide a simpler interpretation of this variable and thereby used one additional cluster to also account for the shot angle: (1) lower part of the paint, (2) upper part of the paint and high post, (3) mid-range wing and corner, (4) three pointers (see Figure 1).

#### Last action before acquiring the ball

This metric provides information about a player’s actions before receiving the ball and eventually attempting a shot. This variable was divided into 11 categories and gives a hint about whether a shot occurred as part of a fast-break or whether a player received the ball as a result of his teammates trying to get him open. The latter case may occur particularly in situations in which a player obtains the ball after coming off a screen or having posted up, or when a spot up or isolation play is run for him.

#### Number of defenders

This metric indicates whether a player is guarded by one, two or three or more defenders at the moment of shot release.

#### Shot defense

This variable captures the defensive intensity against a shooter. We clustered four less relevant categories into one to obtain the following segmentation: (1) Open shots: There is no defender within five feet. (2) Guarded shots: The defender is three to five feet away from the shooter. (3) Pressured shots: The defender is within three feet but does not have his hand raised. (4) Contested shots: The defender is within three feet and raises his hand. (5) Altered shots: The shooter is forced to change either the location of the ball release or the timing. (6) Blocked, goal-tended and fouled shots: These shots are of lesser importance to our analysis since they do not allow unambiguous conclusions about the defensive pressure.

#### Regression analysis

We set up a multiple regression analysis with a stepwise inclusion of predictors, the shot outcome as the dependent variable and all of the above-mentioned factors as independent variables. Since several of the utilized variables are nominally scaled, they were coded into dummy variables for the analysis.

### Results

In the following, we will only present selected results but a more detailed analysis is available in Datasheet S1. The distribution of FG% and the number of field goal attempts (FGA) based on each attribute can be found in Tables 1 and 2. Moreover, we used a one-way ANOVA to test the statistical relevance of each metric and, instead of reporting the results individually, an overview can be found in Table 3.

**Table 1:** Effect of Examined Variables on the FG% and Number of Field Goal Attempts for NBA Players.

Shot type	Dunk/layup	Hook	Floater	Regular jump	Turnaround/fade-away	
FG%FGA	49.4933,338	40.834,014	39.104,639	36.6044,440	34.907,625	

Dribbles	0	1	2	3	4	
FG%FGA	45.0744,959	38.7915,344	37.6111,148	36.565,232	37.7417,373	

Shot clock	≤5 sec.	6–19 sec.	≥20 sec.			
FG%FGA	34.4516,197	41.1566,413	52.1111,446			

Shot location	Lower partof paint	Upper part ofpaint/high post	Mid-range wingand corners	Three pointers		
FG%FGA	46.7342,969	38.6711,698	37.0418,261	35.8720,925		

Pre-ballacqui-sition (1)	Offensiverebound	Steal orloose ball	Transition	Cut	Screen seton ball	Post up
FG%FGA	51.186,051	45.182,605	44.3714,843	42.5312,038	42.417,889	42.2510,460

Pre-ballacqui-sition (2)	Screen setoff ball	Screen receivedoff ball	Defensiverebound	Spot up orisolation	Off doublescreen	
FG%FGA	39.901,020	39.307,733	38.361,533	37.1429,733	36.42151	

**Table 2:** Effect of Defense on the FG% and Number of FGA Based on Shot Location and Number of Statistically Significant Differences (*p*<.05) in Multiple Comparisons.

		Number of defenders			Shot defense type					
Locationcluster		1 defender	2 defenders	3+ defenders	Open	Guarded	Pres-sured	Con-tested	Altered	Block/GT/foul
Lower partof paint	FG%FGASig. comparisons	50.6823,6872	41.8715,711	41.893,5641	91.001,6225	81.251,5735	69.7810,8505	49.5913,9135	43.032,3245	14.6212,6875
Upper part ofpaint/high post	FG%FGASig. comparisons	38.819,6340	37.781,8900	40.801740	45.261,0653	45.111,1663	40.442,1541	39.226,4053	26.67752	13.578334
Mid-rangewingand corners	FG%FGASig. comparisons	37.46 15,8051	34.502,3361	30.831200	44.291,4543	43.031,4783	41.522,9173	37.0011,0675	20.791014	12.621,2444
Threepointers	FG% FGASig.comparisons	35.71 20,241 1	31.69871 1	31.2516 0	41.653,359 4	38.833,101 4	33.612,428 4	34.0911,844 4	3.7027 4	13.82369 4
Total	FG%FGASig. comparisons	41.6569,3674	40.2520,8153	41.463,8741	53.357,50015	49.807,31815	57.0518,34913	40.5843,22917	41.232,52715	14.3815,13317

**Table 3:** Results from One-Way ANOVAs Conducted in Phase 1.

Metric	One-way ANOVA	*p*
Shot type	*F*(4, 94,051) = 372.263**	<.01
Dribbles	*F*(4, 94,051) = 126.749**	<.01
Shot clock	*F*(2, 94,053) = 436.968**	<.01
Shot location	*F*(3, 94,052) = 330.493**	<.01
Pre-ball acquisition	*F*(10, 94,045) = 56.937**	<.01
Number of defendersLower part of paintUpper part of paint/high postMid-range wing and cornersThree pointers	*F*(2, 42,966) = 166.933**F(2, 11,695) = .524*F*(2, 18,258) = 4.821**F(2, 21,125) = 3.013*	<.01.592<.01.049
Shot defenseLower part of paintUpper part of paint/high postMid-range wing and cornersThree pointers	*F*(5, 42,963) = 2,491.334**F(5, 11,692) = 55.132**F(5, 18,255) = 83.881**F(5, 21,122) = 34.681**	<.01<.01<.01<.01

*p<.05 **p<.01.

#### Shot type

FG% for the different categories ranged from 34.90% for fade-away and turnaround jumpers to 49.49% for dunks and layups. Multiple comparisons between shot types yielded significant differences at the 1% level for all comparisons except for two, namely between fade-away and turnaround jumpers and regular jumpers (*p* = .051) as well as floaters and hook shots (*p* = .987).

#### Dribbles

Shots without a dribble featured the highest accuracy at 45.07%, while FG% for shots after two, three and four dribbles were very similar. Multiple comparisons found statistically significant differences for shots which were preceded by no dribble versus one or more dribbles as all of them were significant at the 1% level. Meanwhile, only one other comparison was statistically significant, namely one versus three dribbles (*p* = .046).

#### Remaining time on shot clock

Similar to Skinner [15], we observed a positive relationship with respect to the number of seconds left on the shot clock and FG%. Average shooting percentages ranged from 25.29% to 37.74% in situations where one to five seconds were left, respectively. Meanwhile, by far the highest shooting percentages at 48.04% to 60.81% could be observed with 20 to 24 seconds remaining. In the middle section, i.e., between 6 and 19 seconds, average values ranged from 38.02% to 44.73% and an increasing trend could be observed (see Figure S1). Furthermore, we divided the remaining time into the above-mentioned three sections and multiple comparisons yielded statistically significant differences for all sections at the 1% level.

#### Shot location

In accordance with the findings of Bocskocsky et al. [12], Neiman and Loewenstein [13] and Attali [14], shooting accuracy generally decreased as a function of shot distance and by far the highest FG% could be observed for shots from the lower part of the paint (46.73%). All other shots within the three-point line had a similar average FG% and comparisons yielded significant differences for all clusters at the 5% level.

#### Last action before acquiring the ball

Actions which were a result of teammates trying to get the ball to a player resulted in rather low FG%: spot up or isolation (37.14%) and screen received off the ball (39.30%). The multiple comparisons analysis revealed that most differences between the pre-acquisition actions were not statistically significant. For instance, only one of ten comparisons for the “off double screen” and “screen set off ball” attributes yielded significant differences at the 5% level, respectively.

#### Number of defenders

By far the largest share of shots (74%) was attempted against single coverage. On an aggregate level, it appears as if the number of defenders does not affect the shot outcome, as shots defended by one defender had an average success rate of 41.65%, while shots defended by two (three or more) players had an average FG% of 40.25% (41.46%). However, taking into account shot location, it can be observed that the number of defenders increases as shot distance decreases. We segmented the data based on the number of defenders and the four aforementioned shot location clusters and found that average FG% within each location category tended to decrease the more defenders guarded a shot, as the average decrease in shooting accuracy from being guarded by one to two defenders was 4.21% (see Table 2 and Datasheet S2). This result is intuitive since more players tend to be clustered closer to the basket, so that players attempting a shot from closer distance should generally be surrounded by more defenders than players who attempt a long-distance shot. As illustrated in Table 2, more than 75% (90%) of shot attempts guarded by two (three or more) defenders came from the lower part of the paint, and these distributional differences led to relatively uniform results on an aggregate level (see bottom row of the table). Differences for the multiple comparisons analysis were particularly pronounced in the lower part of the paint and when comparing single versus double coverage.

#### Shot defense

On an aggregate level, pressured shots were made with the highest average FG% followed by open, guarded, altered, and contested shots. However, accounting for shot location, open shots were on average made with the highest average FG% for all four location clusters (see Table 2 and Datasheet S3). Similar to the analysis of the number of defenders, the distribution of the number of shot attempts across the shot defense types varied per shot location cluster and led to different results on an aggregate level. For instance, nearly 60% of all pressured shots were attempted in the lower part of the paint and only 13% of pressured shots came off three pointers, whereas three pointers accounted for roughly 45% of all open shot attempts. Overall, the average decrease in FG% per defense type was 15.28% for shots in the lower part in the paint, while changes ranged from 5.57% to 6.34% for the other location clusters. Results from the multiple comparisons analysis revealed that this variable is a better indicator for defensive pressure than the number of defenders, as all comparisons were relevant for the lower part of the paint and it made a significant difference for most comparisons whether a shot was contested or defended in another way.

#### Regression analysis

The marginal increase in explanatory power of the model quickly became smaller as the number of utilized predictors increased and we used a model with seven predictors because *R^2^* increased by at most .003 per added predictor thereafter. The predictors of the model were found to be – in order of decreasing importance and followed by the associated variable in parentheses – (1) pressured shots (shot defense), (2) open shots (shot defense), (3) contested shots (shot defense), (4) guarded shots (shot defense), (5) lower part of the paint (shot location), (6) altered shots (shot defense), and (7) remaining time on the shot clock. Overall, this model yielded a result of *R^2^* = .113 with *F*(7, 94,048) = 1,708.353 and *p*<.01, while the inclusion of all 18 predictors would have given us a slightly higher explanatory power of *R^2^* = .125. To test the robustness of the model, we used the cross-validation method and divided the dataset into half to run two separate regressions consisting of 47,029 shot attempts each. The results largely matched the ones obtained from the entire dataset, as *R^2^* was found to be .116 (.110) for the first (second) half of the data in the model including seven and .128 (.123) including all predictors. The predictors were also the same for both halves (see Table S1).

### Discussion

As mentioned above, average FG% were lower than in similar NBA datasets because of the inclusion of shots where the shooter was fouled and missed the attempt. For instance, dunks in this dataset have an average FG% of 83.90% but excluding these misses, we obtain a FG% of 89.63%, which is more realistic since dunks rarely result in misses. Furthermore, the shot type analysis provided a good indication about the effect of defensive pressure as turnaround and fade-away shots had the lowest average FG%: If defensive pressure was low, players would not have to fade away or turn around to shoot.

The analysis of the number of dribbles showed that it does not make a significant difference with respect to the shot outcome whether a player dribbles once or several times but a shot attempt without a dribble has a higher chance of being a hit. We hypothesize that players who shoot without a dribble have been put in a good position by their teammates and therefore attempt a less contested shot.

Conversely, shot attempts with a few seconds left on the shot clock are likely a result of high defensive pressure. Specifically, average FG% dipped below 40% for shots attempted with less than 10 seconds on the shot clock. Meanwhile, the results from the shot location analysis were similar to the ones of Bocskocsky et al. [12], Neiman and Loewenstein [13] and Attali [14] as shots from further away were generally associated with a lower FG%.

Regarding the moves before ball acquisition, actions which involved the teammates’ help to get the ball to the eventually shooting player were associated with rather low shooting percentages. These actions likely do not result in an open shot, so that the player has to create his own shot after having received the ball. However, this variable is not a very good indicator of the shot outcome as revealed by the regression and multiple comparisons analyses because most actions do not lead to a significantly different FG% compared to other actions.

Looking at the development of the average FG% relative to the number of defenders, the aggregate results appear striking as they suggest that it does not matter whether players are guarded by one, two or three defenders. The breakdown by shot location reveals that shots from short distance tend to be guarded more intensively, so that the changes in shot difficulty due to distance and defensive pressure offset each other on an aggregate level. Nevertheless, the marginal benefit of an additional defender decreases as the average decrease in FG% from one to two defenders based on the shot location segmentation was 4.21%, while the average FG% actually increased by .26% from two to three defenders. One explanation might be that three defenders stand in each other’s way and do not allow guarding a shot as efficiently. This difference in FG% can also have a significant effect on the outcome of a game: In the 2013–14 NBA season, there were on average 83.0 field goal attempts per game with each hit yielding on average 2.21 points [16]. Therefore, a decrease in accuracy of 4.21% can be translated into 7.72 fewer points allowed per game, which frequently makes the difference between a win and a loss.

Similarly, the shot defense analysis provided some ambiguous results on an aggregate level as pressured shots were hit at a higher rate (57.05%) than open shots (53.35%). Moreover, the data showed the importance of having a hand raised as a defender at the moment of a shot attempt: FG% decreased on average by 6.36% based on the shot location breakdown, while doing so by even 16.47% on an aggregate level relative to pressured shots. As above, we can calculate the effect on a points-per-game basis and a decrease of 16.47% results in 30.21 fewer points per game.

The results of the regression analysis underlined the complexity of basketball as even the inclusion of a wide array of predictors did not lead to a high explanatory power. Furthermore, the model underlined the significance of the shot defense in influencing the outcome of a shot attempt as the four most important predictors belonged to this category. Thus, we will pay special focus in Phase 2 to how the measures in this category develop as a function of a player’s streakiness.

## Phase 2: How Do the Analyzed Metrics Change in Relation to Streakiness?

### Method

Since streak performance has to be assessed on an individual level and a large number of shot attempts is necessary to draw meaningful conclusions, we ran the following analyses with the 26 players who had at least 500 FGA in the dataset (see Datasheet S4 for the list of players and Datasheet S5 for the raw data). In accordance with most of the hot-hand literature and Carlson and Shu [17], who found that people generally needed three repeated events to perceive streakiness, hot (cold) streaks were defined as having hit (missed) at least three consecutive shots. Moreover, we only considered streaks which occurred within a game, so that we excluded the first shot of each game, and neither did we take into consideration misses where the shooter was fouled.

#### Runs test

The runs test is used to count the number of times hits and misses alter in a player’s shooting record, where each string of consecutive misses or hits is considered as a run. Therefore, a player displaying fewer runs than expected by chance can be considered as streaky.

#### Conditional probabilities

Although the remaining game time was not registered in the dataset, shots were coded in chronological order, so that we separately compiled the shooting record of each of the 26 observed players and built an Excel model to calculate the length of each streak. The model identifies the respective player’s next shot following each streak and after filtering the shots for each streak length separately, this shot is used to calculate the players’ performance for the respective streak length. Next, we use *t*-tests to assess whether the FG% of players conditioned on three or more consecutive hits versus misses is statistically different. Furthermore, we use the Durbin-Watson statistic to examine whether a significant serial correlation between streak length and the following shot outcome can be found.

#### Effect of streak length on defensive behavior

We use the above-described Excel model to examine how the players’ shot attempts were distributed within each of the previously presented variables, e.g., what fraction of shot attempts was pressured or contested, before calculating how these proportions changed during hot and cold streaks.

#### Difficulty of streak-ending shots

We analyze the difficulty of shots which end either a hot or a cold streak using various proxies of shot difficulty. Specifically, we compare whether the proportion of difficult streak-ending shots is higher for hot streaks than for cold ones to test whether changes in runs depend on the difficulty of the underlying shot.

#### Effect of hot and cold streaks on FG%

To examine the effects of potential hot-hand behavior, i.e., increases (decreases) in defensive pressure following an opposing player’s hot (cold) streaks, we also analyze how FG% change based on streakiness and the underlying metrics. Specifically, we filter the shooting record of the 26 players based on the various attributes of each variable and the different states of streakiness, i.e., hot, cold and two intermediate states consisting of one or two hits and misses, respectively. Then, we test whether the distribution of FG% during hot and cold streaks is statistically different for selected attributes of each variable.

#### Imperfect streaks consisting of hot (cold) streaks with up to one miss (hit)

As shown above, hot (cold) streaks tended to end with difficult (easy) shot attempts, and a key question about the traditional definition of the hot hand is whether a miss off a difficult FGA is sufficient for a hot streak to be considered as ended in a player’s and an observer’s mind. For instance, should a player on a hot streak, who just missed a difficult turnaround jump shot as the shot clock expired, not be considered as hot anymore? Conversely, is a player’s cold streak nullified if he hits an uncontested dunk on a fast break? To improve the robustness of our results, we expand our previous definition of streakiness by allowing hot (cold) streaks to consist of a miss (hit). Consequently, we examine four-, five- and six-shot sequences, which contain up to one miss and hit for hot and cold streaks, respectively, in the shooting record of the 26 previously observed players. In contrast to the work of Gilovich et al. [2] who divided data into non-overlapping sets of four shots, we separately consider each shot sequence which fulfills the criteria above, so that it is possible for shots attempts to be taken into account more than once in the analysis. For instance, let us assume we are considering five-shot streaks and the underlying streak consists of five consecutive hits followed by a miss. In this case, we coded both the first and last five shots as an (imperfect) hot streak. Following this definition, we run similar analyses to the ones described above and examine how FG% differ for imperfect cold and hot streaks both on an aggregate level and when controlling for shot difficulty. Furthermore, we assess how the share of difficult and easy shots develops for imperfect cold versus hot streaks.

### Results

#### Runs test

The *Z* statistic was positive for 19 of the 26 examined players, so that they exhibited more streaks than expected by chance. However, the results were statistically significant for only one player, namely Chalmers whose data provided evidence for the hot hand (*Z* = 2.242, *p* = .025). Values were negative and non-significant for the remaining players.

#### Conditional probabilities

Across the 26 players, FG% were on average lower during hot (mean: 43.86%) than cold streaks (mean: 49.17%). However, a *t*-test comparing the means of the FG% during hot versus cold streaks found statistically significant differences for only three players, namely Westbrook (*t* = 2.024, *p* = .046), Paul (*t* = 2.416, *p* = .018) and Iguodala (*t* = 2.790, *p*<.01). The Durbin-Watson test yielded negative serial correlations (*d*<2) for 15 players but the *d* statistic was close to 2 and non-significant for all players (see Table 4).

**Table 4:** Shooting Performance During and After Streaks of the 26 NBA Players with the Most FGA in the Dataset.

	Conditional FG%		*t*-test		Runs test			Serial correl.
Player	Hot (≥3 hits)	Cold (≥3 misses)	*t*	*p*	Observed	Expected	*Z*	*d*
L. James	53.29	57.72	–.748	.455	637	651.7	–.817	1.959
K. Durant	47.27	52.38	–.746	.456	552	540.3	.713	1.985
D. Wade	53.00	52.83	.024	.981	515	505.8	.578	2.021
R. Westbrook	31.48	46.90	*–2.024	.046	482	467.7	.945	1.992
K. Bryant	37.25	50.62	–1.513	.133	403	377.6	1.856	2.018
S. Curry	42.11	51.52	–1.039	.301	364	355.4	.643	1.981
J. Harden	52.63	42.86	1.157	.249	344	349.9	–.450	2.015
C. Anthony	43.08	44.93	–.214	.831	356	348.0	.612	2.003
P. Pierce	45.71	50.00	–.427	.670	357	339.9	1.330	1.984
T. Parker	50.00	47.37	.282	.778	349	333.4	1.208	2.013
Z. Randolph	43.18	53.23	–1.015	.313	340	330.5	.740	2.009
K. Garnett	40.35	52.54	–1.314	.191	320	313.0	.562	2.000
B. Griffin	49.21	47.27	.208	.836	308	305.3	.223	1.958
T. Duncan	48.57	58.33	–.948	.345	308	301.5	.531	1.957
R. Gay	51.35	48.48	.277	.783	301	290.9	.842	2.037
C. Paul	30.23	53.13	*–2.416	.018	312	299.8	1.007	1.938
C. Bosh	56.00	39.29	1.729	.087	297	292.8	.345	2.085
R. Rondo	39.13	54.24	–1.541	.126	294	290.7	.278	1.967
M. Conley	40.54	42.65	–.207	.836	263	279.4	–1.403	1.937
A. Iguodala	24.14	53.70	**–2.790	<.01	289	280.2	.747	1.969
D. West	48.00	42.86	.527	.600	270	279.4	–.800	1.944
M. Gasol	46.94	54.35	–.716	.476	267	272.2	–.450	1.919
J. Holiday	53.85	45.33	.858	.393	264	268.7	–.407	2.005
M. Chalmers	27.78	48.08	–1.571	.126	287	261.8	*2.242	1.951
J.R. Smith	41.94	45.33	–.317	.752	246	249.1	–.281	1.874
P. George	43.33	42.42	.083	.934	250	242.2	.727	2.073

*p<.05 **p<.01.

#### Effect of streak length on defensive behavior

In general, the observed players tended to attempt shots which were associated with a lower average FG% (as shown in Phase 1) during hot streaks, and vice versa during cold streaks (see Figure S2). The proportion of the shot type with the lowest mean FG%, i.e., fade-away and turnaround jumpers, increased from 3.13% for shots following seven consecutive misses to 26.92% for shots after seven consecutive hits across the 26 players, whereas the share of dunks and layups, which is the shot type with the highest mean FG%, showed a decreasing trend.

Meanwhile, the fraction of shot attempts without a dribble stayed relatively constant regardless of whether a player was on a hot or cold streak. Specifically, shot attempts without a dribble constituted about 30 to 40% of all shot attempts, with small outliers coming following cold streaks.

Changes as a function of streak length were more pronounced when examining the number of remaining seconds on the shot clock. For streaks of six and seven consecutive misses, the average remaining time was over 12 seconds before the curve flattened out for the following streaks lengths. Finally, a drop below 11 and 10 seconds occurred for streaks of 6 and 7 consecutive hits, respectively.

Furthermore, our results indicate that the position from which shots were attempted is affected by streak length. Specifically, the share of shots from the lower part of the paint decreased as 49.60% of all FGA came from this section on average during cold streaks but only 36.90% did so during hot streaks.

Concerning the pre-ball acquisition move, we investigated the three categories which are the most indicative of whether a player is put in a good position to receive the ball. Players tended to receive slightly more screens off the ball as a function of streak length and receive the ball more frequently after posting up but changes were very subtle (mean change: .89% for screens received and 1.41% for post up). Meanwhile, changes in spot up or isolation plays as a function of streak length were very volatile and did not yield meaningful results.

With respect to the number of defenders, the share of shot attempts defended by one player increased fairly steadily until a streak length of four consecutive hits before dropping sharply from 79.48% to 57.69% for streaks of seven hits. Conversely, the share of shot attempts defended by two or three players increased for long hot streaks.

Finally, analyzing the development of the different defense types shows that the share of open shots steadily decreased from 18.75% during cold streaks to 3.08% and 3.85% during hot streaks. Meanwhile, the share of pressured shots increased from 3.13% for streaks of seven misses to 23.08% for streaks of seven hits but values were around 20% for most streak lengths. Lastly, with the exception of an outlier for streaks of seven consecutive misses, the share of contested shots steadily rose from 41.67% to 61.54% as streak length increased.

#### Difficulty of streak-ending shots

In accordance with the findings above, the results suggest that hot streaks tended to end with the miss of a relatively difficult shot, whereas players frequently hit a relatively easy shot to end cold streaks (see Table 5 for an overview of the results for the most relevant attributes). For instance, 58.10% of cold streaks ended with a relatively easy shot attempt from the lower part of the paint, whereas only 28.57% of hot streaks did so. In contrast, 25.71% (11.58%) of streaking-ending shots came from three-point range for hot (cold) streaks. Similarly, the share of cold streaks which ended as a result of an open shot was nearly twice as large as that of hot streaks (10.25 versus 5.32%), whereas roughly 11% more hot streaks ended following a contested shot compared to cold streaks.

**Table 5:** Analysis of Difficulty of Streak-Ending Shots Based on Selected Attributes.

		Hot (≥3 hits)		Cold (≥3 misses)		
Metric	Attribute	FGA	% of FGA	FGA	% of FGA	Δ hot - cold
Shot type	Dunk/layup	132	17.14%	421	46.42%	–29.27%
	Hook	25	3.25%	32	3.53%	–.28%
	Floater	46	5.97%	61	6.73%	–.75%
	Regular jump	446	57.92%	312	34.40%	23.52%
	Turnaround/fade-away	121	15.71%	81	8.93%	6.78%
Dribbles	0	261	33.90%	341	37.60%	–3.70%
	≥1	509	66.10%	566	62.40%	3.70%
Shot	≤5 seconds	185	24.03%	138	15.21%	8.81%
clock	6–19 seconds	545	70.78%	642	70.78%	.00%
	≥20 seconds	40	5.19%	127	14.00%	–8.81%
Shot location	Lower part of paint	220	28.57%	527	58.10%	–29.53%
	Upper part ofpaint/high post	146	18.96%	124	13.67%	5.29%
	Mid-range wingand corners	206	26.75%	151	16.65%	10.10%
	Three pointers	198	25.71%	105	11.58%	14.14%
Pre-ball acquisition move	Offensive rebound	18	2.34%	61	6.73%	–4.39%
	Steal/loose ball	26	3.38%	27	2.98%	.40%
	Transition	143	18.57%	219	24.15%	–5.57%
	Post up	95	12.34%	111	12.24%	.10%
	Screen received off ball	73	9.48%	76	8.38%	1.10%
	Spot up/isolation	264	34.29%	210	23.15%	11.13%
	Off double screen	0	.00%	2	.22%	–.22%
Number of defenders Shot defense	1	596	77.40%	637	70.23%	7.17%
	2	161	20.91%	234	25.80%	–4.89%
	3+	13	1.69%	36	3.97%	–2.28%
	Open	41	5.32%	93	10.25%	–4.93%
	Guarded	66	8.57%	61	6.73%	1.85%
	Pressured	140	18.18%	224	24.70%	–6.51%
	Contested	444	57.66%	421	46.42%	11.25%
	Altered	25	3.25%	34	3.75%	–.50%
	Block/GT/foul	54	7.01%	74	8.16%	–1.15%

#### Effect of hot and cold streaks on FG%

**Figure 2:**
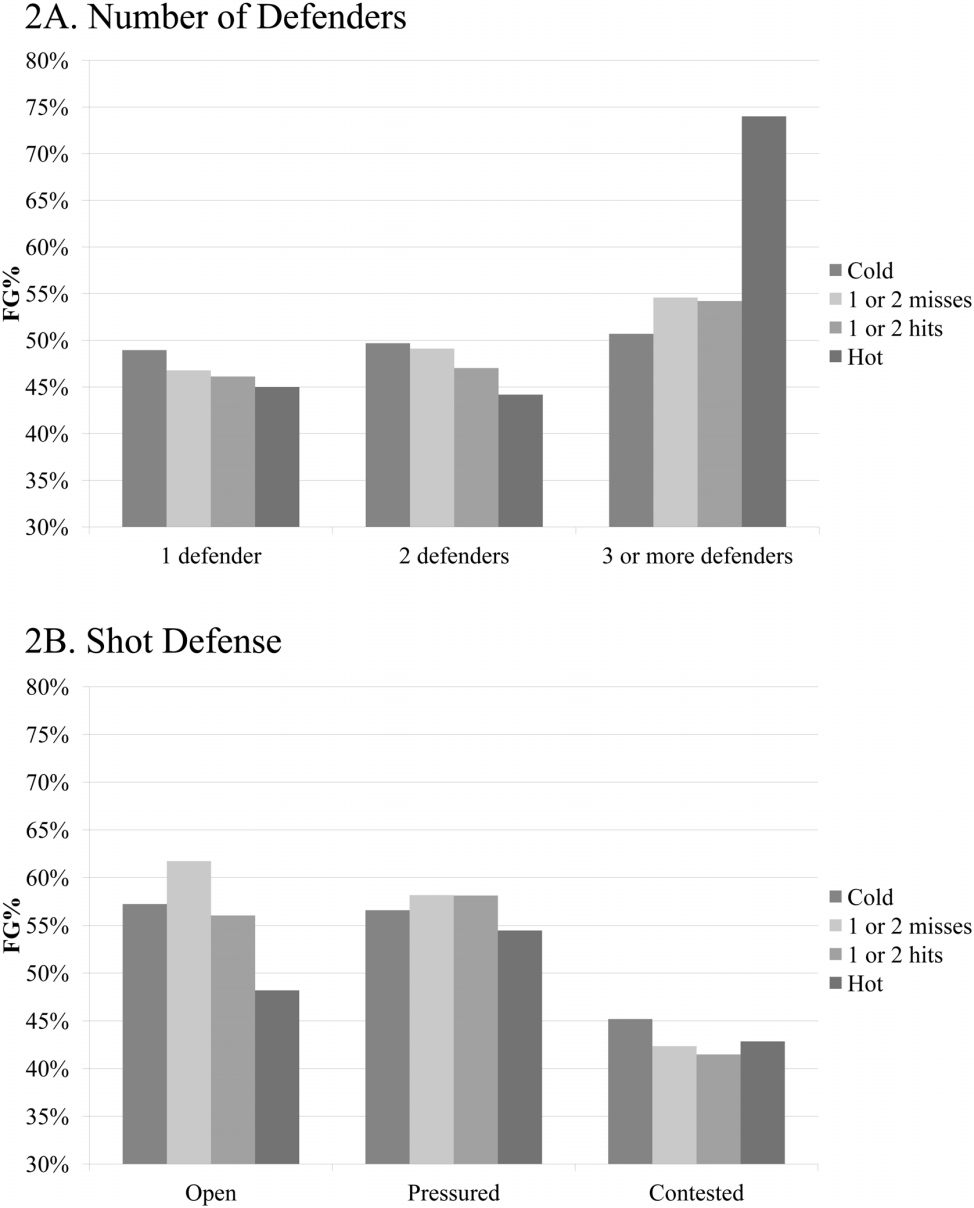
Evolution of FG% Conditional on the Number of Consecutive Hits and Misses and Selected Defensive Metrics. 2A. Number of Defenders. 2B. Shot Defense.

Overall, the analysis allowed the observation of only very few trends in the data. For most variables, differences in shooting percentages were small with observed values frequently being lower for hot than cold states (see Figure S3). The *t*-test yielded significant differences for only one of 19 comparisons, namely for shots defended by three or more players. In this case, FG% increased from 50.70% during cold to 74.00% during hot streaks. In contrast, a decreasing trend in FG% from cold to hot streaks could be observed for shots defended by one and two players, respectively (see Figure 2A).

Similarly, shooting percentages were lower during hot than cold streaks when the data is segmented based on the different shot defense types. The largest decrease could be observed for open shots as FGA during hot streaks were converted with a 9.04% lower accuracy compared to cold streaks (see Figure 2B). For pressured and contested shots, this difference shrunk to 2.12% and 2.34%, respectively.

#### Imperfect streaks consisting of hot (cold) streaks with up to one miss (hit)

Overall, the results are in line with the ones from the analysis in which only perfect streaks were considered but the magnitude of changes declined in many cases (see Table 6 for an overview). For instance, when comparing the FG% of the 26 observed players, shooting accuracies were on average lower after hot streaks relative to cold ones for four-, five- and six-shot sequences but differences were not as large as for perfect streaks (see Table 4).

**Table 6:** Analysis of Imperfect Streaks Including 1 Miss (Hit) for Hot (Cold) Streaks.

	4 FGA			5 FGA			6 FGA		
	3–4 hits	3–4 misses	Δ hot - cold	4–5 hits	4–5 misses	Δ hot - cold	5–6 hits	5–6 misses	Δ hot - cold
FG%	46.49%	47.05%	–.56%	45.77%	47.36%	–1.59%	45.99%	46.06%	–.07%
*Share of total FGA*									
Dunks/layups	27.33%	32.94%	–5.61%	25.77%	32.54%	–6.76%	24.68%	31.93%	–7.25%
Turnaround/fade-away	13.99%	12.69%	1.30%	13.84%	12.06%	1.78%	14.57%	12.26%	2.31%
Shot clock <6 seconds	19.76%	21.25%	–1.49%	20.50%	22.01%	–1.50%	21.61%	20.84%	.76%
Shot clock >19 seconds	8.75%	10.70%	–1.96%	8.46%	10.49%	–2.03%	8.72%	10.07%	–1.35%
Lower part of paint	37.51%	44.40%	–6.89%	35.69%	43.64%	–7.96%	33.60%	42.78%	–9.18%
Three pointers	20.88%	18.89%	1.98%	21.12%	19.82%	1.30%	22.79%	20.30%	2.50%
1 defender	74.25%	72.05%	2.20%	75.69%	71.64%	4.05%	76.02%	72.99%	3.03%
2 defenders	22.11%	24.13%	–2.02%	20.67%	24.28%	–3.61%	19.23%	22.79%	–3.57%
3+ defenders	3.64%	3.82%	–.18%	3.64%	4.09%	–.45%	4.76%	4.22%	.54%
Open shots	6.85%	7.64%	–.79%	6.83%	8.05%	–1.22%	7.53%	8.20%	–.66%
Contested shots	54.32%	52.93%	1.39%	54.85%	52.11%	2.74%	55.70%	52.85%	2.85%
*FG% conditional on difficulty*									
Dunks/layups	64.49%	61.10%	3.39%	64.41%	62.90%	1.51%	66.98%	61.52%	5.46%
Turnaround/fade-away	37.30%	38.63%	–1.33%	36.44%	37.33%	–.89%	36.43%	35.67%	.77%
Shot clock <6 seconds	32.51%	37.97%	–5.46%	31.69%	35.71%	–4.02%	31.87%	35.34%	–3.47%
Shot clock >19 seconds	61.59%	64.67%	–3.08%	63.76%	64.17%	–.41%	61.64%	65.12%	–3.47%
Lower part of paint	57.67%	57.82%	–.15%	57.80%	59.43%	–1.64%	59.20%	58.97%	.22%
Three pointers	37.77%	34.67%	3.11%	36.87%	36.04%	.83%	36.41%	35.77%	.64%
1 defender	44.83%	46.99%	–2.16%	43.77%	46.81%	–3.04%	42.73%	44.83%	–2.10%
2 defenders	49.67%	46.91%	2.77%	49.32%	48.46%	.86%	47.16%	50.00%	–2.84%
3+ defenders	62.99%	50.30%	12.69%	69.23%	52.53%	16.71%	75.61%	50.00%	25.61%
Open shots	56.54%	53.75%	2.79%	53.72%	50.52%	3.20%	50.00%	52.88%	–2.88%
Contested shots	40.84%	43.39%	–2.55%	40.63%	43.45%	–2.82%	39.29%	41.92%	–2.63%

Regarding the development of shot difficulty as a function of streakiness, the share of difficult shot attempts tended to be much higher during imperfect hot streaks than during cold ones. Specifically, the share of FGA coming off easy shot types, i.e., layups and dunks, was 5.61 to 7.25% lower during hot streaks (depending on the length of the observed shot sequence), while the share of turnaround and fade-away jump shots increased by 1.30 to 2.31% during hot streaks. A more pronounced trend could be observed when analyzing different shot location clusters, as 6.89 to 9.18% fewer FGA came from the lower part of the zone during imperfect cold streaks compared to hot ones. Similarly, the share of contested shots was higher (1.39 to 2.85% increase) during imperfect hot streaks, while players attempted fewer (.66 to 1.22% decrease) open shots.

Finally, when analyzing how shooting percentages developed as a function of shot difficulty and streakiness, FG% during imperfect cold streaks were mostly higher than ones during hot streaks but this trend could be observed less frequently and its magnitude was smaller than in the analysis of only perfect streaks. For instance, dunks and layups were on average actually hit with a higher accuracy of 1.51 to 5.46% following imperfect hot streaks compared to cold ones, and the same held for three pointers. With respect to the variable measuring defensive intensity, open shots were on average hit with a 2.79% (3.20%) higher accuracy for imperfect hot sequences consisting of four (five) shots, whereas the mean FG% was 2.88% lower when looking at six-shot hot streaks. Furthermore, shooting accuracies for contested shots were on average 2.55 to 2.82% lower following imperfect hot streaks.

### Discussion

The goal of Phase 2 was to examine whether defenders were prone to display hot-hand behavior and whether this behavior can be considered adaptive. The results of the traditional tests, i.e., the runs test and the analysis of conditional probabilities, were in line with the literature as no significant evidence for the hot hand could be found. The runs tests indicated that one player exhibited more streaks than expected by chance, while the *t*-test yielded significant differences for three players. Given a sample size of 26 players, these results do not provide sufficient evidence for the hot hand. Similarly, one of nine players in the analysis of Gilovich et al. [2] had a statistically significant *Z* score.

When assessing the evolution of the share of shot attempts as a function of streak length for different shot types and shot locations, we find that players attempted more difficult (easier) shots as the length of hot (cold) streaks increased. Meanwhile, other variables, such as the number of dribbles, were mostly unaffected by whether a player was on a hot or cold streak or whether he did not experience any streakiness at all. A similar trend could be observed for the three selected pre-ball acquisition moves as only slight increases in FGA coming off “screen received off ball” and “post up” took place as streak length increased. Furthermore, the number of observations became relatively small for longer streaks since the pre-ball acquisition move encompassed more attributes than other variables. With respect to the remaining time on the shot clock, a fairly sharp drop occurred for shots with five seconds or less on the shot clock during hot streaks exceeding five consecutive hits (15.86% for five versus 30.77% seven consecutive hits), which suggests a tendency to give the ball to a hot player in situations where time is expiring.

As indicated in Phase 1, the results emphasize that the analysis of the number of defenders was not as meaningful as that of the defensive intensity. More pronounced changes for this variable could only be observed for hot streaks following four or more consecutive hits. The trend of higher shot difficulty with increasing streak length became more visible when looking at how the shot defense types evolved. With the exception of streaks of seven consecutive misses, the share of pressured shots mostly stayed constant regardless of streak length, while the share of contested shots increased steadily. Combining these findings with the way the different defense types affected shooting percentages, it seems as if defenders did not increase the pressure by a small increment from “open” to “pressured” during hot streaks but that they did so in a more intensive way by contesting shots. In general, the results for long streaks, i.e., consisting of five or more consecutive shots, have to be interpreted with caution because they were experienced fairly rarely by the observed players. While we believe that the statistical power of the results was generally sufficient due to the large database and the large number of FGA of the observed players, future research should try to validate our results for long hot and cold streaks using a more extensive database.

The analysis of streak-ending shots yielded results which are in line with the findings above as most hot streaks tended to end following difficult shots, whereas cold streaks did so after relatively easy shots. Since it was previously shown that players attempted a larger share of difficult (easy) shots during hot (cold) streaks, it is not surprising that streaks end in such a way since the observed shots are a subset of the analysis above. However, this measure may provide an alternative to the runs test as it also examines the potential reasons for a change in runs. Moreover, these results are of relevance to athletes as they indicate that hot players might be better off to pass up difficult shot attempts and wait for easier ones to come around because they are likely to end a hot streak.

In sum, there is a tendency for players to attempt more difficult (easier) shots following several consecutive hits (misses) and for defenders to behave according to the hot-hand belief. The question arising from this finding is whether the defenders’ behavior can be considered as ecologically rational, i.e., whether increased defensive pressure can be justified by hot players hitting shots with higher accuracy. A key assumption which is needed to assess the ecological rationality is that such strategic moves usually entail some kind of tradeoff, e.g., they come at the cost of leaving other players open (in accordance with Aharoni & Sarig [10]). Based on this assumption, our results indicate that the observed behavior cannot be classified as ecologically rational as the shooting performance was not elevated based on the breakdown by the different attributes of each metric. Instead, FG% tended to be slightly lower during hot than cold streaks, which is in line with most of the literature (e.g., Gilovich et al. [2]). As mentioned, players on hot streaks were much more prone to miss open shots, so defenders might be better off not increasing the defensive pressure even if an opposing player has hit several consecutive shots. The only case where the FG% was significantly higher during hot streaks relative to cold streaks was for shots which were defended by three or more players. One explanation for the strong increase in FG% might be the small sample size: As shown in Table 2, only 4.12% of all shot attempts came against three defenders and the observed FG% of 74.00% came off 50 shots whereas the other results came off several hundred observations. To provide a more definite answer about the ecological rationality of hot-hand behavior by defenders, a fruitful avenue for future research could be to relate our findings to the overall performance of a team during a player’s hot phases. For instance, it could be examined based on a measure similar to the plus-minus statistic whether the team with a hot player tends to outscore the opponent in phases in which the performance of the player in question is elevated.

The analysis of imperfect streaks confirmed the trend that shooting percentages were generally lower during hot streaks compared to cold ones and that shot difficulty tended to increase (decrease) during hot (cold) streaks, thereby providing more solid evidence for our findings. However, the magnitude of the difference in mean shooting accuracies became smaller compared to perfect streaks, so that our results about the non-existence of the hot-hand phenomenon and the non-adaptiveness of hot-hand behavior have to be treated with caution. For instance, open shots were hit with a 9.04% lower accuracy during hot streaks when only taking into account perfect streaks but these shots were actually hit at a higher rate when we examined four- and five-shot streaks with at most one miss. Therefore, the ability to detect the hot-hand effect may depend on the underlying assumptions and definitions of the hot-hand phenomenon. Further research should investigate whether the miss of a difficult shot – or a hit of an easy layup in the case of a cold streak – is truly sufficient to end a streak in both the concerned player’s as well as the observer’s perception. Moreover, the statistical approach may have influenced the results of our work since we did not run any simulations with the dataset and a model analysis would provide further insights about the potential existence of the hot hand.

## Conclusions

The current work presented a multitude of novel performance metrics in the light of the hot-hand phenomenon and directly examined the impact of defensive pressure on the hot hand for the first time. We find that the “shot defense” metric serves as the best proxy for defensive intensity. Previous research examining the effect of streakiness on the shot selection of NBA players claimed that the hot hand in basketball was unobservable because of the game structure and the opposing team’s reaction. Similar to the findings of Aharoni and Sarig [10], our results indicate that shot difficulty indeed increases (decreases) following hot (cold) streaks and this is a result of defenders increasing their pressure. However, when looking at how FG% evolves as a breakdown of the respective variables, our analysis indicates that a player’s performance is not elevated during hot streaks.

Aharoni and Sarig [10] examined whether shooting percentages were statistically different during hot streaks versus normal states. While they showed that differences were not statistically significant, average FG% were lower during hot streaks and the authors did not provide a more detailed breakdown of how shooting percentages evolved relative to the increased shot difficulty. Similarly, we found no statistically significant differences in FG% for most players for cold versus hot streaks but the additional analysis revealed that shooting percentages tended to be lower during hot streaks when accounting for shot difficulty. In contrast to our work, Bocskocsky et al. [12] estimated the hot hand to result in an increased FG% of .53% but their dataset did not include the defensive intensity, i.e., whether a defender raised his hand to contest a shot. Instead, their proxy of defensive pressure was mostly based on the location of defenders and defender distance, which was measured from the center of the respective players’ body mass, so that appendages, such as a raised hand, were not accounted for, and the authors acknowledged that this was “a clear deficiency” (p. 9) of their dataset. As shown in our dataset, shots against three or more defenders are indeed hit with an overall higher FG% during hot streaks but the relevance of this result is questionable due to the rare occurrence of shots being guarded by three defenders during hot streaks and the finding that FG% did not differ by more than 1.40% on an aggregate level regardless of whether shots were defended by one, two or three players. Meanwhile, our analysis of both perfect and imperfect streaks showed that the players’ performance decreased during hot streaks when considering contested shots. A fruitful avenue for future research would be to reconcile our findings regarding the existence of the hot hand with the ones of Bocskocsky et al. [12] and examine reasons for the partially different results.

A deficiency of the dataset is that it did not include the time at which each shot was attempted, so that we could not provide a more detailed breakdown by analyzing the effect of the time interval between shots within a game on a player’s streakiness. Although we believe that an important factor of streakiness is that consecutive shots occur within a reasonable timeframe, we hypothesize based on previous hot-hand research that such an additional analysis would not have yielded significantly different conclusions: Firstly, Adams [18] hypothesized that it is more likely that a shot results in a hit if less time has elapsed since the preceding hit but the results actually indicated that the opposite was the case, namely that time intervals were shorter when a hit was followed by a miss. Secondly, Aharoni and Sarig [10] examined the streakiness of NBA players by restricting hot streaks to a halftime of a game, i.e., a player had to hit at least three consecutive shots in a half to be considered hot. According to this definition, the authors found that the FG% of hot players was 1.80% lower during hot streaks compared to their average, whereas the shooting accuracy of the observed players in our dataset was on average 3.10% lower than the base rate. Therefore, we believe that this difference of 1.30% would not have led to significantly different conclusions even if we had been able to provide a more detailed analysis of the effect of the time interval between shot attempts.

Furthermore, it has frequently been hypothesized in the literature (e.g., Burns [8], Willer, Sharkey, & Frey [19]) that teammates try to “feed” the hot player and put him in a good position to score but this possibility could previously not be examined due to a lack of metrics. We tested this hypothesis using three selected pre-ball acquisition moves and our results do not provide evidence that this is the case: Players did not receive significantly more screens off the ball, isolation or post up plays after several consecutive shots and the observed difference, if any, was only very small. Relating our findings to the concept of ecological rationality, defenders appear to increase the defensive pressure on a player who has hit several consecutive shots because they believe that this player has a higher chance of scoring. Since players do not appear to possess an elevated performance level during hot streaks – in particular when considering perfect streaks – the defenders’ behavior cannot be classified as ecologically rational based on our findings. Instead, they would likely be better off applying less pressure on hot players and equally focusing on the other four players.

In the current work, we have exclusively focused on the behavior displayed by professional players in game situations. While our results indicate that defensive strategies in the NBA are shaped by the hot-hand belief and that results about the existence of the hot hand depend on the underlying definition of the phenomenon, a fruitful avenue for further research could be to explore how changes in shot difficulty as well as imperfect streaks shape the belief in the hot hand. For instance, is it possible that a player who has hit two highly contested turnaround jumpers is considered to be “hotter” than a player who has hit four open jump shots? Can a player still be labeled as hot after having missed a tightly contested three pointer? Observed increases in shot difficulty during hot streaks may potentially explain why the belief is so widespread although actual performance increases cannot be observed. Furthermore, future research should be directed to a more detailed analysis using simulations to increase the statistical power of our findings and provide further insights into the existence of the hot-hand phenomenon.

## Supporting Information

Figure S1 **Effect of the Remaining Seconds on the Shot Clock on the FG%.**
(TIF)

Figure S2 **Changes of Different Variables as a Function of Streak Length.** Figure S2A. Shot Types. Figure S2B. Shot Attempts without a Dribble. Figure S2C. Seconds on the Shot Clock. Figure S2D. Seconds on the Shot Clock (Clustered View). Figure S2E. Shot Location Clusters. Figure S2F. Pre-Ball Acquisition Moves. Figure S2G. Number of Defenders. Figure S2H. Defense Types.
(TIF)

Figure S3 **Evolution of FG% Based on Hot and Cold Streaks for Different Variables.** Figure S3A. Shot Types. Figure S3B. Number of Dribbles. Figure S3C. Remaining Seconds on the Shot Clock. Figure S3D. Shot Location Clusters. Figure S3E. Pre-Ball Acquisition Moves.
(TIF)

Table S1 **Results of Regression Model Predicting the Shot Outcome Based on 7 Predictors.**
(DOCX)

Datasheet S1 **Overview Results of Shooting Metrics.**
(XLSX)

Datasheet S2 **Number of Defenders and Location Breakdown.**
(XLSX)

Datasheet S3 **Shot Defense and Location Breakdown.**
(XLSX)

Datasheet S4 **List of Players with 500 or More FGA.**
(XLSX)

Datasheet S5 **Raw Data Used for Analysis in Phase 2.**
(XLSX)
