# Correction: "Hand down, Man down." Analysis of Defensive Adjustments in Response to the Hot Hand in Basketball Using Novel Defense Metrics

**DOI:** 10.1371/journal.pone.0124982

**Published:** 2015-04-13

**Authors:** Peter Csapo, Markus Raab

There is an error in the first sentence of the "Imperfect streaks consisting of hot (cold) streaks with up to one miss (hit)" portion of the “Method” subsection of the "Phase 2: How Do the Analyzed Metrics Change in Relation to Streakiness?" section. The correct sentence is: As hypothesized above, hot (cold) streaks might end with difficult (easy) shot attempts, and a key question about the traditional definition of the hot hand is whether a miss off a difficult FGA is sufficient for a hot streak to be considered as ended in a player’s and an observer’s mind.

Table 1 has been corrected for improved readability. Please see the corrected Table 1 here.

**Table 1:** Effect of Examined Variables on the FG% and Number of Field Goal Attempts for NBA Players.

Shot type	Dunk/ layup	Hook	Floater	Regular jump	Turnaround/fade-away	
FG%	49.49	40.83	39.10	36.60	34.90	
FGA	33,338	4,014	4,639	44,440	7,625	
**Dribbles**	0	1	2	3	4	
FG%	45.07	38.79	37.61	36.56	37.74	
FGA	44,959	15,344	11,148	5,232	17,373	
**Shot clock**	≤ 5 sec.	6–19 sec.	≥ 20 sec.			
FG%	34.45	41.15	52.11			
FGA	16,197	66,413	11,446			
**Shot location**	Lower part of paint	Upper part of paint/ high post	Mid-range wing and corners	Three pointers		
FG%	46.73	38.67	37.04	35.87		
FGA	42,969	11,698	18,261	20,925		
**Pre-ball acquisition (1)**	Offensive rebound	Steal or loose ball	Transition	Cut	Screen set on ball	Post up
FG%	51.18	45.18	44.37	42.53	42.41	42.25
FGA	6,051	2,605	14,843	12,038	7,889	10,460
**Pre-ball acquisition (2)**	Screen set off ball	Screen received off ball	Defensive rebound	Spot up or isolation	Off double screen	
FG%	39.90	39.30	38.36	37.14	36.42	
FGA	1,020	7,733	1,533	29,733	151	

Table 2 has been corrected for improved readability. Please see the corrected Table 2 here.

**Table 2:** Effect of Defense on the FG% and Number of FGA Based on Shot Location and Number of Statistically Significant Differences (*p*<.05) in Multiple Comparisons.

		Number of defenders			Shot defense type					
Location cluster		1 defender	2 defenders	3+ defenders	Open	Guarded	Pressured	Contested	Altered	Block/ GT/foul
Lower part of paint	FG%	50.68	41.87	41.89	91	81.25	69.78	49.59	43.03	14.62
	FGA	23,687	15,718	3,564	1,622	1,573	10,850	13,913	2,324	12,687
	Sig. comparisons	2	1	1	5	5	5	5	5	5
Upper part of paint/ high post	FG%	38.81	37.78	40.8	45.26	45.11	40.44	39.22	26.67	13.57
	FGA	9,634	1890	174	1,065	1,166	2,154	6,405	75	833
	Sig. comparisons	0	0	0	3	3	1	3	2	4
Mid-range wing and corners	FG%	37.46	34.5	30.83	44.29	43.03	41.52	37	20.79	12.62
	FGA	15,805	2,336	120	1,454	1,478	2,917	11,067	101	1,244
	Sig. comparisons	1	1	0	3	3	3	5	4	4
Three pointers	FG%	35.71	31.69	31.25	41.65	38.83	33.61	34.09	3.7	13.82
	FGA	20,241	871	16	3,359	3,101	2,428	11,844	27	369
	Sig. comparisons	1	1	0	4	4	4	4	4	4
Total	FG%	41.65	40.25	41.46	53.35	49.8	57.05	40.58	41.23	14.38
	FGA	69,367	20,815	3,874	7,500	7,318	18,349	43,229	2,527	15,133
	Sig. comparisons	4	3	1	15	15	13	17	15	17

Table 3 has been corrected for improved readability. Please see the corrected Table 3 here.

**Table 3:** Results from One-Way ANOVAs Conducted in Phase 1.

Metric	One-way ANOVA	*p*
Shot type	*F*(4, 94,051) = 372.263**	< .01
Dribbles	*F*(4, 94,051) = 126.749**	< .01
Shot clock	*F*(2, 94,053) = 436.968**	< .01
Shot location	*F*(3, 94,052) = 330.493**	< .01
Pre-ball acquisition	*F*(10, 94,045) = 56.937**	< .01
Number of defenders		
Lower part of paint	*F*(2, 42,966) = 166.933**	< .01
Upper part of paint / high post	*F*(2, 11,695) = .524	.592
Mid-range wing and corners	*F*(2, 18,258) = 4.821**	< .01
Three pointers	*F*(2, 21,125) = 3.013*	.049
Shot defense		
Lower part of paint	*F*(5, 42,963) = 2,491.334**	< .01
Upper part of paint / high post	*F*(5, 11,692) = 55.132**	< .01
Mid-range wing and corners	*F*(5, 18,255) = 83.881**	< .01
Three pointers	*F*(5, 21,122) = 34.681**	< .01

* *p* < .05

** *p* < .01

Table 5 has been corrected for improved readability. Please see the corrected Table 5 here.

**Table 5:** Analysis of Difficulty of Streak-Ending Shots Based on Selected Attributes

		Hot (≥ 3 hits)			Cold (≥ 3 misses)		
Metric	Attribute	FGA	% of FGA	FGA		% of FGA	Δ hot—cold
Shot type	Dunk/ layup	132	17.14%	421		46.42%	-29.27%
	Hook	25	3.25%	32		3.53%	-.28%
	Floater	46	5.97%	61		6.73%	-.75%
	Regular jump	446	57.92%	312		34.40%	23.52%
	Turnaround/ fade-away	121	15.71%	81		8.93%	6.78%
Dribbles	0	261	33.90%	341		37.60%	-3.70%
	≥ 1	509	66.10%	566		62.40%	3.70%
Shot clock	≤ 5 seconds	185	24.03%	138		15.21%	8.81%
	6–19 seconds	545	70.78%	642		70.78%	.00%
	≥ 20 seconds	40	5.19%	127		14.00%	-8.81%
Shot location	Lower part of paint	220	28.57%	527		58.10%	-29.53%
	Upper part of paint/ high post	146	18.96%	124		13.67%	5.29%
	Mid-range wing and corners	206	26.75%	151		16.65%	10.10%
	Three pointers	198	25.71%	105		11.58%	14.14%
Pre-ball acquisition move	Offensive rebound	18	2.34%	61		6.73%	-4.39%
	Steal/ loose ball	26	3.38%	27		2.98%	.40%
	Transition	143	18.57%	219		24.15%	-5.57%
	Post up	95	12.34%	111		12.24%	.10%
	Screen received off ball	73	9.48%	76		8.38%	1.10%
	Spot up/ isolation	264	34.29%	210		23.15%	11.13%
	Off double screen	0	.00%	2		.22%	-.22%
Number of defenders	1	596	77.40%	637		70.23%	7.17%
	2	161	20.91%	234		25.80%	-4.89%
	3+	13	1.69%	36		3.97%	-2.28%
Shot defense type	Open	41	5.32%	93		10.25%	-4.93%
	Guarded	66	8.57%	61		6.73%	1.85%
	Pressured	140	18.18%	224		24.70%	-6.51%
	Contested	444	57.66%	421		46.42%	11.25%
	Altered	25	3.25%	34		3.75%	-.50%
	Block/ GT/ foul	54	7.01%	74		8.16%	-1.15%
