# Supplementary figures and images for: “Hand down, Man down.” Analysis of Defensive Adjustments in Response to the Hot Hand in Basketball Using Novel Defense Metrics
Source: PLoS One. 2014 Dec 4;9(12):e114184. doi: 10.1371/journal.pone.0114184 (PMC4256225; doi:10.1371/journal.pone.0114184)

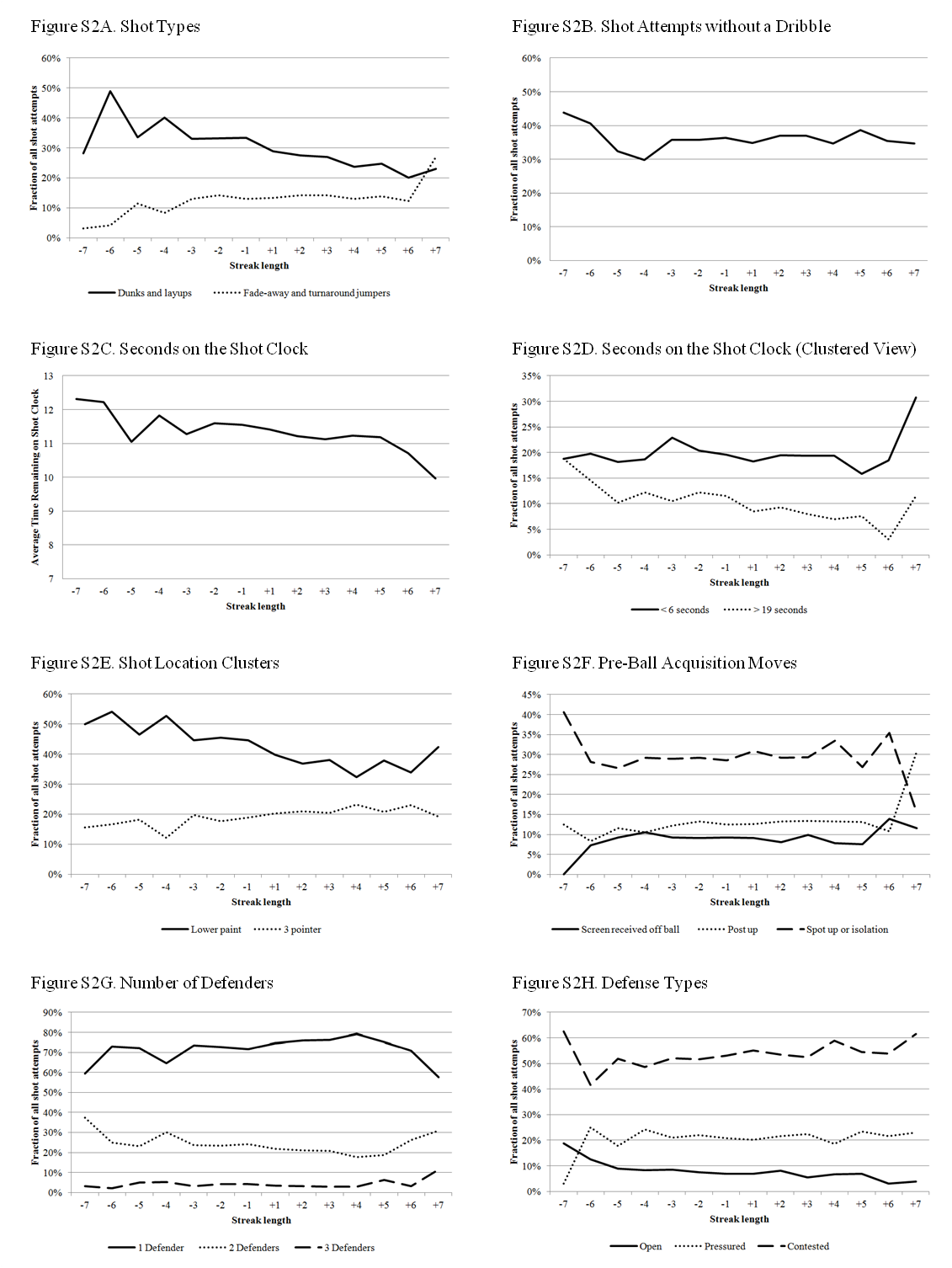

Supplement: Figure S2 — Changes of Different Variables as a Function of Streak Length. Figure S2A. Shot Types. Figure S2B. Shot Attempts without a Dribble. Figure S2C. Seconds on the Shot Clock. Figure S2D. Seconds on the Shot Clock (Clustered View). Figure S2E. Shot Location Clusters. Figure S2F. Pre-Ball Acquisition Moves. Figure S2G. Number of Defenders. Figure S2H. Defense Types. (TIF) [file pone.0114184.s002.tif]

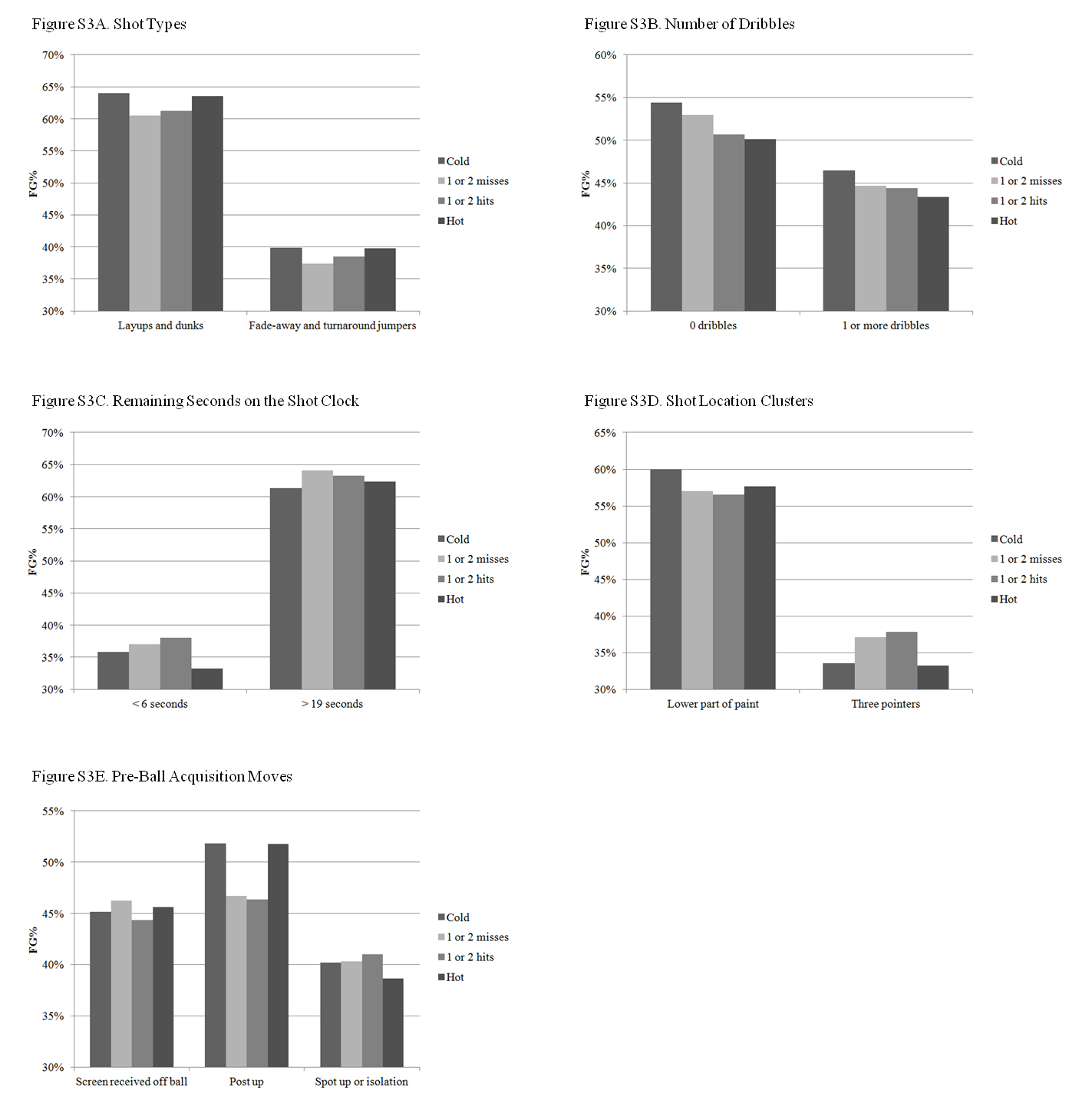

Supplement: Figure S3 — Evolution of FG% Based on Hot and Cold Streaks for Different Variables. Figure S3A. Shot Types. Figure S3B. Number of Dribbles. Figure S3C. Remaining Seconds on the Shot Clock. Figure S3D. Shot Location Clusters. Figure S3E. Pre-Ball Acquisition Moves. (TIF) [file pone.0114184.s003.tif]
