# Supplementary material for: “Hand down, Man down.” Analysis of Defensive Adjustments in Response to the Hot Hand in Basketball Using Novel Defense Metrics
Source: PLoS One. 2014 Dec 4;9(12):e114184. doi: 10.1371/journal.pone.0114184 (PMC4256225; doi:10.1371/journal.pone.0114184)
Supplement: Table S1 — Results of Regression Model Predicting the Shot Outcome Based on 7 Predictors. (DOCX) [file pone.0114184.s004.docx]

**Table S1. Results of Regression Model Predicting the Shot Outcome Based on 7 Predictors**

| Predictor | Unstand-  ardized *β* | *t*-test | *p* |
| --- | --- | --- | --- |
| Constant | -.079 | **-14.496 | < .01 |
| Pressured shots | .473 | **91.593 | < .01 |
| Open shots | .508 | **73.592 | < .01 |
| Contested shots | .369 | **78.248 | < .01 |
| Guarded shots | .476 | **68.498 | < .01 |
| Lower part of the paint | .190 | **54.623 | < .01 |
| Altered shots | .255 | **25.600 | < .01 |
| Shot clock | .005 | **18.861 | < .01 |

* *p* < .05 ** *p* < .01
